# Identification of serum miR-1915-3p and miR-455-3p as biomarkers for breast cancer

**DOI:** 10.1371/journal.pone.0200716

**Published:** 2018-07-26

**Authors:** Jian Guo, Chen Liu, Wei Wang, Yan Liu, Huiwen He, Chong Chen, Rong Xiang, Yunping Luo

**Affiliations:** 1 Department of Immunology, Institute of Basic Medical Sciences Chinese Academy of Medical Sciences, School of Basic Medicine Peking Union Medical College, Beijing, China; 2 Collaborative Innovation Center for Biotherapy, Institute of Basic Medical Sciences Chinese Academy of Medical Sciences, School of Basic Medicine Peking Union Medical College, Beijing, China; 3 Department of Hematology, The First Affiliated Hospital of Chongqing Medical University, Chongqing, China; 4 Collaborative Innovation Center for Biotherapy, Medical College of Nankai University, Tianjin, China; University of North Carolina at Chapel Hill School of Medicine, UNITED STATES

## Abstract

Breast cancer is one of the most malignant diseases in women worldwide. Serum microRNAs (miRNAs), with the characteristics of high sensitivity and specificity, have recently attracted more attentions to serve as potential biomarkers for tumor diseases. In this study, 194 breast cancer patients’ serum samples were collected before surgery and enrolled into different groups based on their diagnostic information. To search for breast cancer diagnostic biomarkers, serum miRNAs were screened by microarray in pooled samples of healthy volunteers and breast cancer patients in different clinical stages. The miRNAs were further verified in each individual patient’s serum samples in diagnostic and predictive sets. The serum level of miR-1915-3p was upregulated and miR-455-3p was downregulated significantly in breast cancer patients compared with healthy volunteers. Furthermore, the patients with infiltrating carcinoma or lymph node metastasis had a higher serum level of miR-1915-3p and lower serum level of miR-455-3p than patients with the carcinoma in situ or patients without lymph node metastasis. ROC analysis suggested that miR-1915-3p and miR-455-3p had the potential as a promising serum diagnostic and predictive biomarkers of breast cancer. miR-1915-3p was over-expressed in certain human breast cancer cells. Functional experiments in vitro showed that miR-1915-3p enhanced cell proliferative and migrational abilities. Overexpression of miR-1915-3p repressed target gene DUSP3 and activated ERK1/2. Collectively, this study provided a new insight that miR-1915-3p might play a role in the development of breast cancer and that serum miR-1915-3p and miR-455-3p could serve as diagnostic and predictive biomarkers for breast cancer.

## Introduction

Breast cancer is one of the most malignant cancers in women worldwide. According to the latest statistical report in China, the age-standardized rate (ASR) of newly-diagnosed breast cancer was 21.6 cases per 100,000 women, making breast cancer the most common cancer in Chinese women[1]. It was reported that breast cancer is the second leading cause of cancer death in women younger than 45 years of age. The largest proportion of new cancer cases and deaths among women are diagnosed among those between the ages 60 and 74 years[2]. However, the 5-year survival of early-stage breast cancer could be 78~80%. Therefore, the earlier that breast cancer can be diagnosed and treated, the longer survival could be.

microRNAs, which were found highly conserved in most eukaryotic organisms, could repress protein translation in post transcriptional regulation way and influence the biological functions of cells[3, 4]. Abnormal expressed miRNAs have been proved to be the significant character in many types of cancer, which could perform their carcinogenesis influence or pathological process regulating function in many existing ways, including intracellular and extracellular miRNAs[5, 6]. More and more evidences have supported that circulating miRNAs have the potential to help clinical diagnosis and prognosis analysis[7, 8]. With the advantages of stability and sensitivity, serum miRNAs has been emerging as promising sensitive and specific biomarkers for early detection of cancers and many other serious diseases. Therefore, serum miRNAs are becoming one of the most popular research subjects in molecular diagnostics[9–11].

In this study, we focused on serum miRNAs and screened breast cancer patients serum abnormal expressed miRNAs by using Affymetrix genechip. The results were confirmed by qRT-PCR. We selected specific miRNAs through statistical analysis to identify diagnostic and predictive markers of breast cancer and explored the potential mechanisms.

## Materials and methods

### Study design, patients description and control individuals

The study population consisted of 94 breast cancer patients (mean age, 48 years; range, 28–86 years) and 100 patients with benign tumor (mean age, 39.8 years; range, 19–66 years) who didn’t receive any direct or specific treatment after being enrolled in the study. The control population included 100 healthy volunteers. Controls were matched to the patients based on age, gender and ethnicity. The patients were all newly diagnosed and had not received any treatment for cancer prior to blood collection. The breast cancer patients were subdivided into two groups in two ways: one was lymph nodes metastasis (n = 36) or non-metastasis (n = 58), and the other was carcinoma in situ (n = 25) or infiltrating carcinoma (n = 69). We collected both breast cancer patients’ and healthy volunteers’ serum from January 2015 to October 2015. Serum was aliquoted and stored at -80°C until used. A multiphase, case-control study was designed to identify serum miRNAs as surrogate markers for breast cancer. Initially, we subjected seven pooled serum samples from malignant, benign, infiltrate carcinoma, carcinoma in situ, lymph node positive and negative cases and health matched controls to perform miRNAarray by using Affymetrix GeneChip, which was aiming at identifying abnormal serum miRNAs in breast cancer cases when compared with controls. Tumor tissue histopathological analysis was performed according to the WHO criteria. Patients were eligible if they had been diagnosed with a pathological breast cancer that met histological or cytological criteria. The tumor stage at the time of diagnosis was assessed according to the American Joint Committee on Cancer guidelines. To validate the biomarkers for diagnosis and prediction, the study was carried out in two sets: (a) diagnostic set, including 90 serum samples: 30 breast cancer patients (include 10 carcinoma in situ, 20 infiltrate carcinoma, 15 lymph node positive and 15 lymph node negative patients), 30 patients with benign tumor and 30 healthy control; and (b) predictive set, consisting of 64 serum samples: 15 patients who were diagnosed with carcinoma in situ, 49 with infiltrate carcinoma, 21 with lymph node positive and 43 with lymph node negative. This study has been approved by INSTITUTIONAL REVIEW BOARD of Institute of Basic Medical Sciences, Chinese Academy of Medical Sciences. Informed consents of patients and healthy volunteers were obtained by written consent.

### Serum preparation and extraction of total RNA

Venous blood samples(~ 5ml) were collected from each patient and healthy volunteer and no one had received any treatment for cancer prior to blood collection. Total RNA was isolated from serum by acid-phenol, chloroform and acidic sodium, and the precipitation was carried out by ethanol. The extracted RNA was eluted in 30ul DEPC water and measured on a NanoDrop ND-1000 Spectrophotomete(Thermo Scientific). The RNA samples were immediatedly stored at -80°C until use.

### Reverse transcription PCR and quantitative real-time PCR

The miRNA-specific TaqMan miRNA Assays (Applied Biosystems) for 11 candidate miRNAs were used according to manufacture’s protocol. 2ul of total RNA was reverse transcribed using primers specific in each of the miRNA target. After reverse transcription, qRT-PCR were performed in a LightCycler® 480 (Roche Diagnostics) with 20ul reaction volumes containing 1ul reverse transcription product, 0.33ul Taqman probe, 2ul 10X buffer, 0.4ul dNTP, 0.3ul Taq DNA polymerase and 15.97ul H_2_O. All experiments were done in duplicate. The mimics of each miRNAs(ordered from Guangzhou Ruibo, China) were used as a control to calculate the absolute concentration. The reactions were incubated in 96-well plates at 95°C for 5min, followed by 40 cycles of amplication (95°C for 15s, 60°C for 60s).

### Cell culture and transfection

MDA-MB-231 cell line was purchased from ATCC and SUM-159 cell line was a gift from Dr. Peiqing Sun (WFU, USA). Boh cells were cultured in high glucose Dulbecco’s Modified Eagle Medium(DMEN)(Invitrogen) supplemented with 10% fetal bovine serum (FBS) (Invitrogen), penicillin, and streptomycin. Transfection was performed by using lipo2000 solution and Opti-MEM culture medium according to the manufacturer’s protocols.

### Cell migration assay

5x10^4^ MDA-MB-231 or SUM-159 cells were seeded on a upper transwell chamber (8.0 μm pore, Corning) in a 24 well plate. After 24 hours, the cells on top of the insert membrane were removed. The cells that had migrated to the bottom of the insert membrane were fixed and stained with crystal violet, followed by light microscope imaging.

### Cell colony formation assay

Cells were seeded into a 6-well plate in duplicate wells at a density of 400 cells/well. The culture media was refreshed every 48 hours. Approximately on day 7, the old media was removed with a pipette, and the cells were gently rinsed two times with PBS. Cells were then fixed with methanol and stained with hematoxylin overnight. The staining solution was slowly washed with flowing water. An inverted microscope was used to observe the number of clone formations of each cell.

### CCK8 assay

Cells were seeded into each of the 96 wells at a density of 5x10^4^cells/well. CCK-8 solution was added to each well and then the absorbance at 450nm was measured with a microplate reader after incubation for two hours. The cell growth was measured for five day continuously.

### 5-ethynyl-2′-deoxyuridine (EdU) proliferation assay

Cells were seeded into a 24-well plate for 72h, and then studied with a Cell-Light™ EdU Apollo®488 In Vitro Imaging Kit according to the manufacturer's instructions (RiboBio). Images were taken and analyzed using a Confocal FV1000 microscope (Olympus). Percentages of EdU-positive cells were calculated as follows: (EdU-positive cells/Hoechst stained cells) × 100%. At least 200 cells were counted per well.

### Dual-luciferase report system assays

The wild type or mutant 3’UTR sequences of DUSP3 were cloned into the psiCHECK2 plasmid (Promega). HEK-293FT cells were co-transfected with 300ng of recombinant plasmid and 50nmol miR-1915-3p or negative control by Lipofectamine 2000(Invitrogen) in 24-well plates. Luciferase activities were detected with a Dual-Luciferase Reporter Assay Kit (Promega) 24h later.

### Western blot

Total proteins were obtained by the protein extraction reagent (Pierce). Protein samples were loaded in NuPAGE gels (Invitrogen) and transferred to PVDF membrane (Bio-rad). After blocked with 5%BSA, the membranes were incubated with primary antibodies overnight at 4°C. The membranes were washed three times in TBST for 10min and incubated with secondary antibodies for 2h at room temperature. Then the membranes were washed in TBST as before. Lastly, the protein bands on membranes were examined by Western ECL Substrate (Bio-rad) and the images were caught by Tanon-5800 Chemiluminescent Imaging System (Tanon).

### Data analysis

For qPCR results, software supplied with Roche LightCycler480 was used for analyzing absolute concentrations. The different concentration of miRNA mimics standards (10pmol/μl, 10^-2^pmol/μl, 10^-3^pmol/μl, 10^-4^pmol/μl, 10^-5^pmol/μl and 10^-6^pmol/μl.) were used to draw the standard curve. Based on Ct values of standards, second order derivative spectrophotometry was used to measure the absolute concentrations of miRNAs in each sample. The wells were in duplicate. The average concentrations were imported into SPSS 21.0 to draw the statistical chart, which resulted in a ROC curve. The confidence interval was defined at 95%. The statistical information was shown in S1 File.

## Results

### Affymetrix miRNA profiling array results

**Fig 1:**
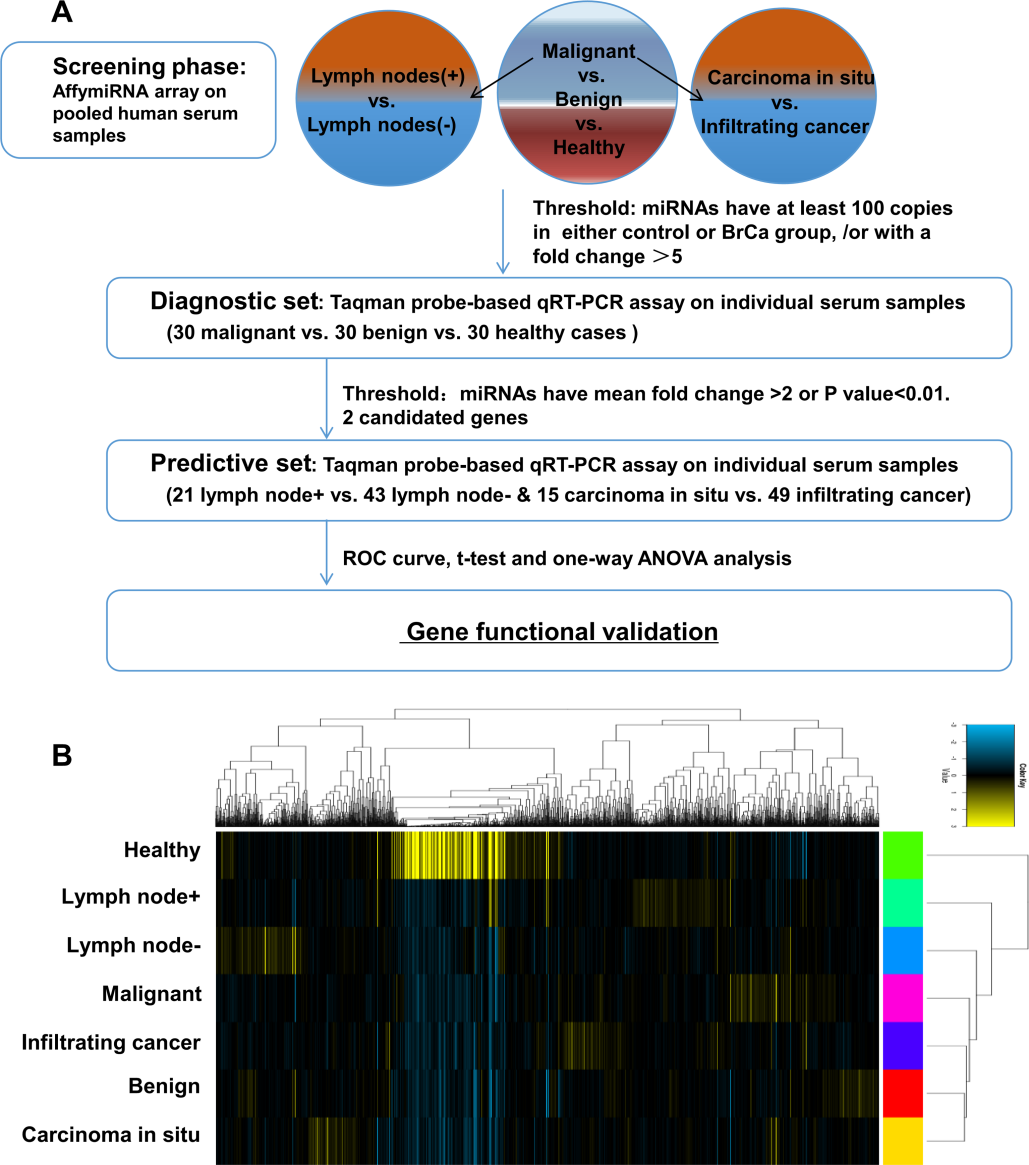
Differential expression of serum miRNAs in the breast cancer patients and healthy volunteers. (A). Flow chart of the experimental design. (B). Cluster analysis of serum miRNA on pooled serum samples.

As shown in the flow chart (Fig 1A), the experiment was generally divided into three parts. As mentioned before, seven groups of patients’ serum samples were pooled together respectively, and screened by Affymetrix miRNA microarray. The results of the microarray were submitted on ArrayExpress (accession number: E-MTAB-6652). All the patients’ diagnostic information was categorized in S1 Table. The copy reads of individual serum miRNAs were measured in seven groups’ pooled sample. The different expressed miRNAs in malignant v.s. benign v.s. healthy groups, lymph node (+) v.s. lymph node (-) groups, and carcinoma in situ v.s. invasive carcinoma groups were shown in Fig 1B, and cluster analysis was performed based on the whole results of microarray. Through comparison between breast cancer, breast fibroadenoma and healthy persons, with the referenced filter criteria (more than 5-fold change between groups or copy reads greater than 100), nine different expressed miRNAs (miR-455-3p, miR-638, miR-1825, miR-1915-3p, miR-3613-5p, miR-4487, miR-6821-5p miR-5001-5p and miR-8075) were identified. The expression value of each miRNA in different groups was listed in Table 1.

**Table 1:** Selected genes microarray raw data.

Transcript ID (Array Design)	Malignant	Benign	Healthy	Lymph node-	Lymph node+	Carcinoma in situ	Infiltrate carcinoma
hsa-miR-455-3p	104.0	7.8	4.1	11.1	5.3	63.9	5.2
hsa-miR-638	283.0	320.2	1564.2	25.1	441.2	66.9	185.8
hsa-miR-1825	19.6	3.7	3.7	5.2	5.2	4.6	9.1
hsa-miR-1915-3p	81.1	12.8	1123.7	11.2	72.2	36.3	14.4
hsa-miR-3613-5p	409.5	561.9	12.0	42.6	17.3	268.6	820.3
hsa-miR-4487	315.2	88.4	9.3	95.6	164.4	87.9	135.9
hsa-miR-6749-5p	52.8	7.6	81.6	12.9	18.4	43.5	49.3
hsa-miR-8075	906.4	903.5	76.6	623.8	1061.1	407.0	1164.8
hsa-miR-5001-5p	98.4	6.6	59.5	25.0	91.0	8.3	32.7

### Validation of expression profiles for serum miRNAs serving as diagnostic biomarkers of breast cancer

**Fig 2:**
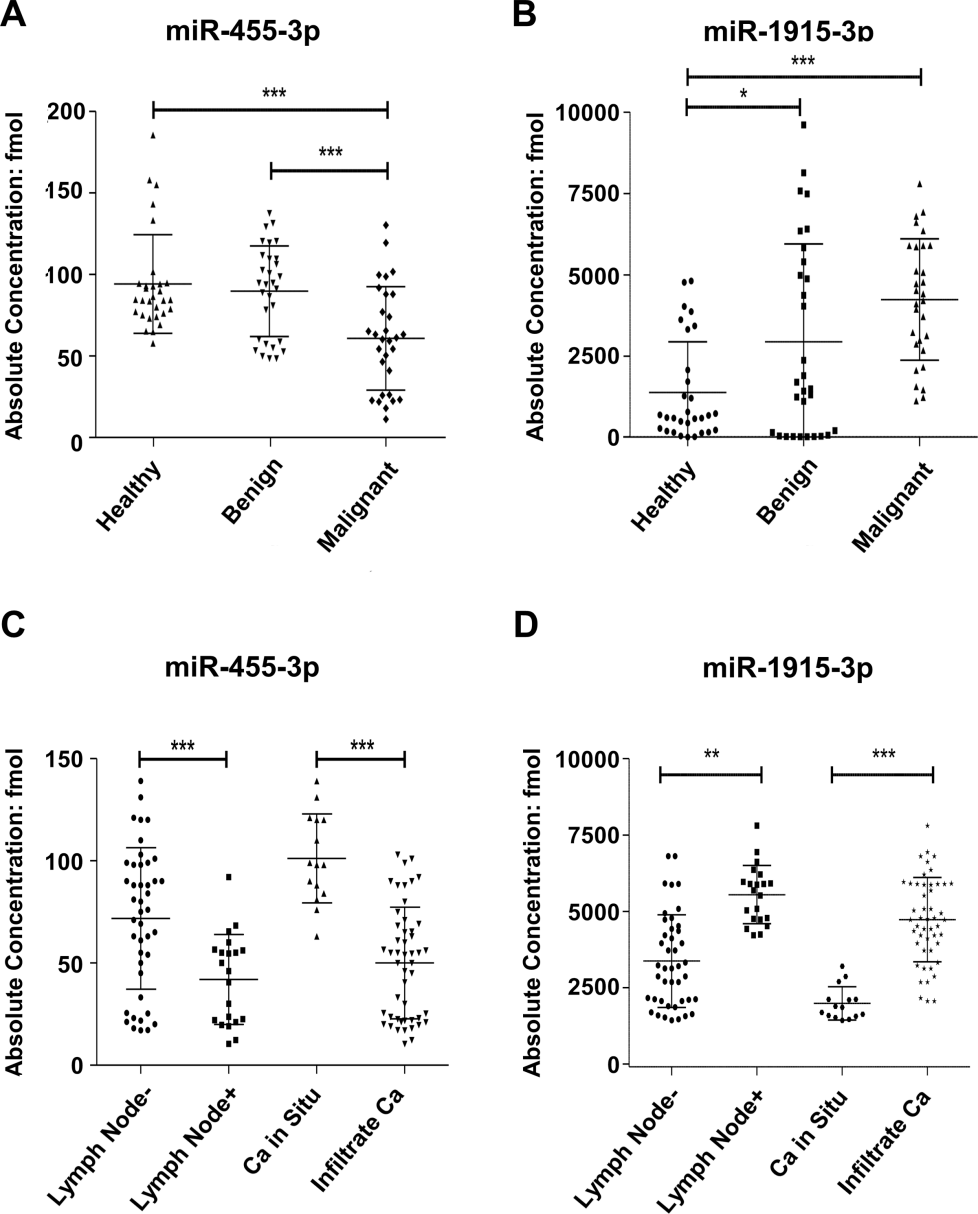
miR-455-3p or miR-1915-3p levels of all single serum sample in the diagnostic set and predictive set. Serum levels of the miR-455-3p and miR-1915-3p were measured in (A,B) diagnostic set (thirty malignant, thirty benign and thirty healthy cases) and (C,D) predictive set (21 lymph node+ and 43 lymph node-, as well as 15 carcinoma in situ and 49 infiltrating cancer) by using Taqman probe RT-qPCR assay. (*P<0.05, **P<0.01, ***P<0.001).

Thirty breast cancer samples, thirty fibroadenoma samples and thirty healthy samples were selected to fill the diagnostic set and the single miRNA expression level was detected by TaqMan Probe. One-way ANOVA was used to analyze the statistic significance of serum miRNAs expression level in each group. However, to our surprise, the validation results were not in accordance with the array results. Even so, miR-1915-3p and miR-455-3p still showed diagnostic value on breast cancer. Scatter diagrams were respectively prepared for these two miRNAs with their absolute concentrations. As shown in Fig 2A and 2B, miR-1915-3p and miR-455-3p had statistically differential expressions in healthy group, benign group and malignant group. miR-1915-3p has a higher expression in the benign group and the malignant group, while lower expression in the healthy group. Meanwhile miR-455-3p has a lower expression in the malignant group as compared with the healthy group and benign group. The other seven miRNAs expression values were shown in S1 Fig.

The predictive set included 64 cases of breast cancer (including 43 cases of lymph node non metastasis, 21 cases of lymph node metastasis, 15 cases of carcinoma in situ and 49 cases of invasive carcinoma). As shown in Fig 2C and 2D, the serum concentration of miR-1915-3p had a higher expression in the breast invasive carcinoma group and lymph node metastasis group than that of the carcinoma in situ group and lymph node non metastasis group, while miR-455-3p has lower expression in breast invasive carcinoma group and lymph node metastasis group than in carcinoma in situ group and lymph node non metastasis group. Taken together, these results demonstrated that serum miR-1915-3p and miR-455-3p had statistically differential expressions in both sets and might serve as diagnostic biomarkers for breast cancer.

### ROC curve analysis

**Fig 3:**
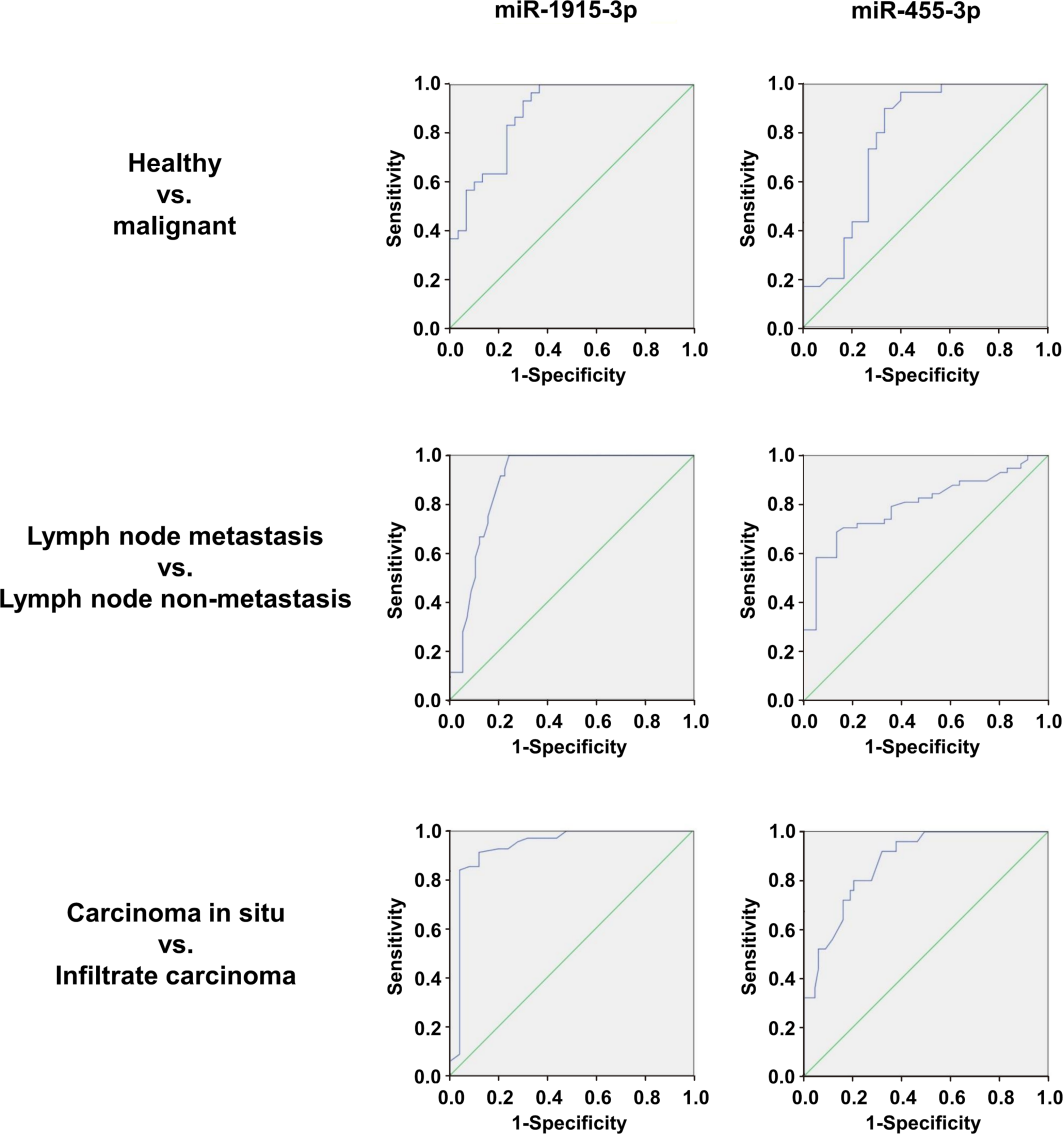
Diagnostic performance of serum miR-455-3p and miR-1915-3p for breast cancer. ROC curve analysis for the two miRNAs was performed to differentiate malignant from healthy groups; lymph node metastasis from non metastasis groups; carcinoma in situ group from infiltrating carcinoma group.

ROC(receiver-operating characteristic) was plotted to compare the miRNAs serum concentrations in all samples of both the diagnostic and the predictive sets. ROC curve is a well recognized statistical method and is widely used for identifying diseases predictive accuracy. The horizontal axis represents the specificity and the vertical axis represents the sensitivity. The Area Under Curve (AUC) represents the accuracy of predicted results. The larger the AUC is (close to 1), the more accurate the prediction will be. As shown in the Fig 3, ROC curve analysis comparing the expression level of the two miRNAs for malignant and healthy groups yielded the following AUCs: miR-455-3p, 0.778(95%CI: 0.654–0.9020); miR-1915-3p, 0.881(95%CI: 0.797–0.965). For lymph node metastasis and non metastasis groups, the ROC curve analysis yielded the following AUCs: miR-455-3p, 0.795 (95%CI: 0.705–0.886); miR-1915-3p, 0.894 (95%CI: 0.829–0.960). For carcinoma in situ group and infiltrating carcinoma group, we also performed ROC curve analyses on the selected miRNAs and obtained the respective AUCs: miR-455-3p, 0.876 (95%CI: 0.806–0.946); miR-1915-3p, 0.934 (95%CI: 0.845–1.000). This data indicated that both of the two miRNAs exhibited reliable performance in diagnosing breast cancer and predicting lymph node metastasis and infiltrating status.

### miR-1915-3p promotes the proliferation of MDA-MB-231 and SUM-159 cells

**Fig 4:**
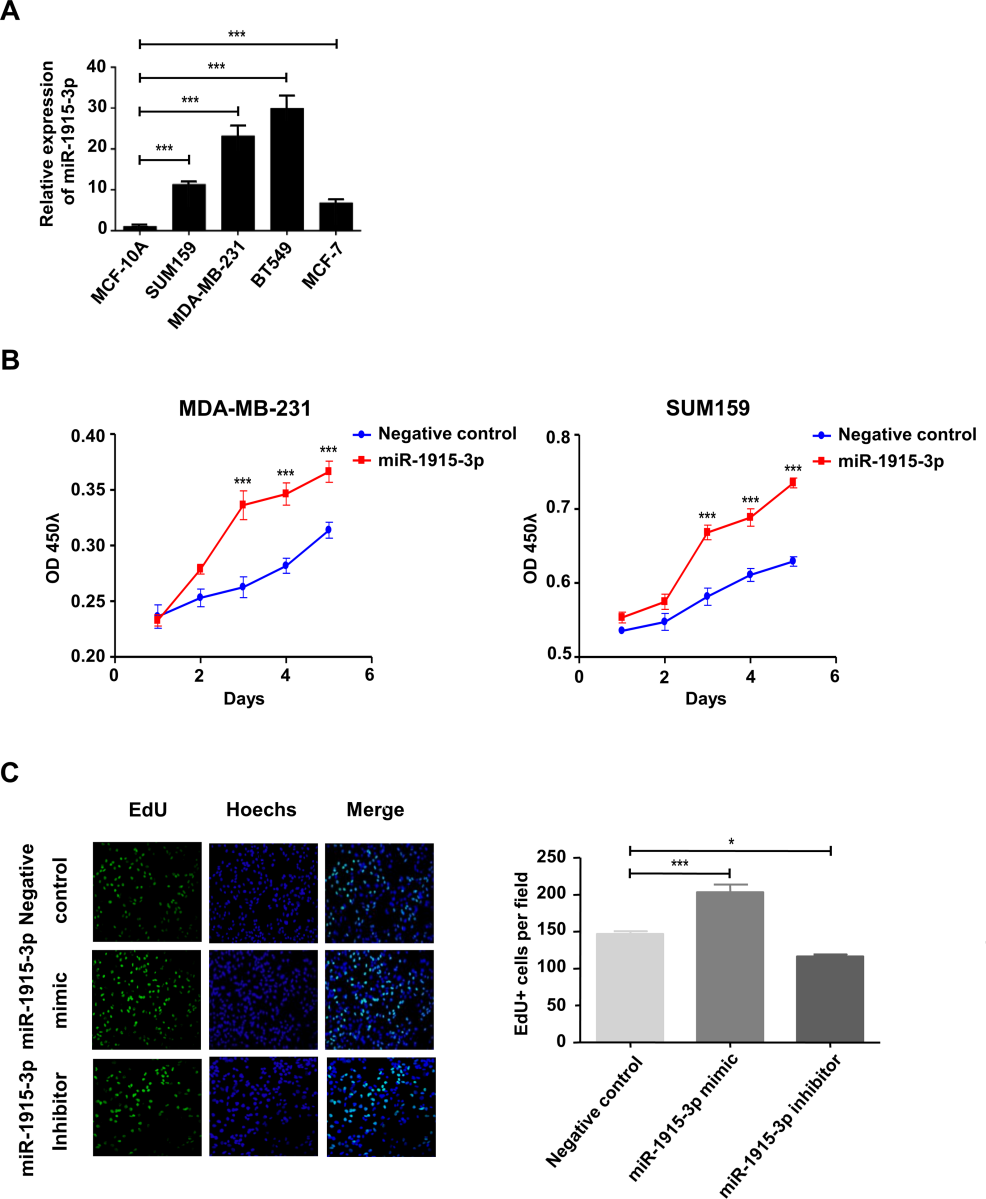
miR-1915-3p promoted breast cancer cell proliferation in vitro. (A) miR-1915-3p expression in breast cancer cells were measured by Taqman probe RT-qPCR assay. (B) CCK8 assay was performed on both MDA-MB-231 and SUM-159 cells which were transfected with miR-1915-3p mimics (red line) or negative control (blue line). OD450 value was measured every 24 hours during 5 days culture. (C) The EdU staining (Green) of MDA-MB-231 cells transfected with miR-1915-3p mimic or inhibitor was performed after seeding cells 72 hours, meanwhile cell nucleus were stained with Hochest (Blue), statistic result was shown on the right. Data was shown as mean±s.e.m. from three independent experiments (***P<0.001,**P<0.01, *P<0.05).

miRNA expression is frequently dysregulated in cancer. Abnormal high-level expression of miRNA in tumor, secreting into the blood both actively and passively, is one of the most important reason for increased serum level of miRNA[12]. miR-1915-3p was over-expressed in certain human breast cancer cells (Fig 4A). MDA-MB-231 and SUM-159 are common human breast cancer cell lines. Both cell lines have appropriate proliferation ability and migration ability and are suitable for in vitro functional experiments, also mechanism validation. To further investigate the influence of miR-1915-3p on breast cancer cell proliferation, we overexpressed miR-1915-3p in MD-MBA-231 and SUM-159 cell lines, and then the cell proliferation ability was detected by using a CCK-8 assay. It showed that miR-1915-3p could significantly promote cell proliferation (Fig 4B).

The EdU assay was then conducted to further confirm their influence on cell proliferation imposed by transfecting miR-1915-3p mimics and inhibitors in MDA-MB-231 cells. As shown in Fig 4C, the percentage of cells in S-phase was significantly increased after over-expression and decreased after silencing of miR-1915-3p.

**Fig 5:**
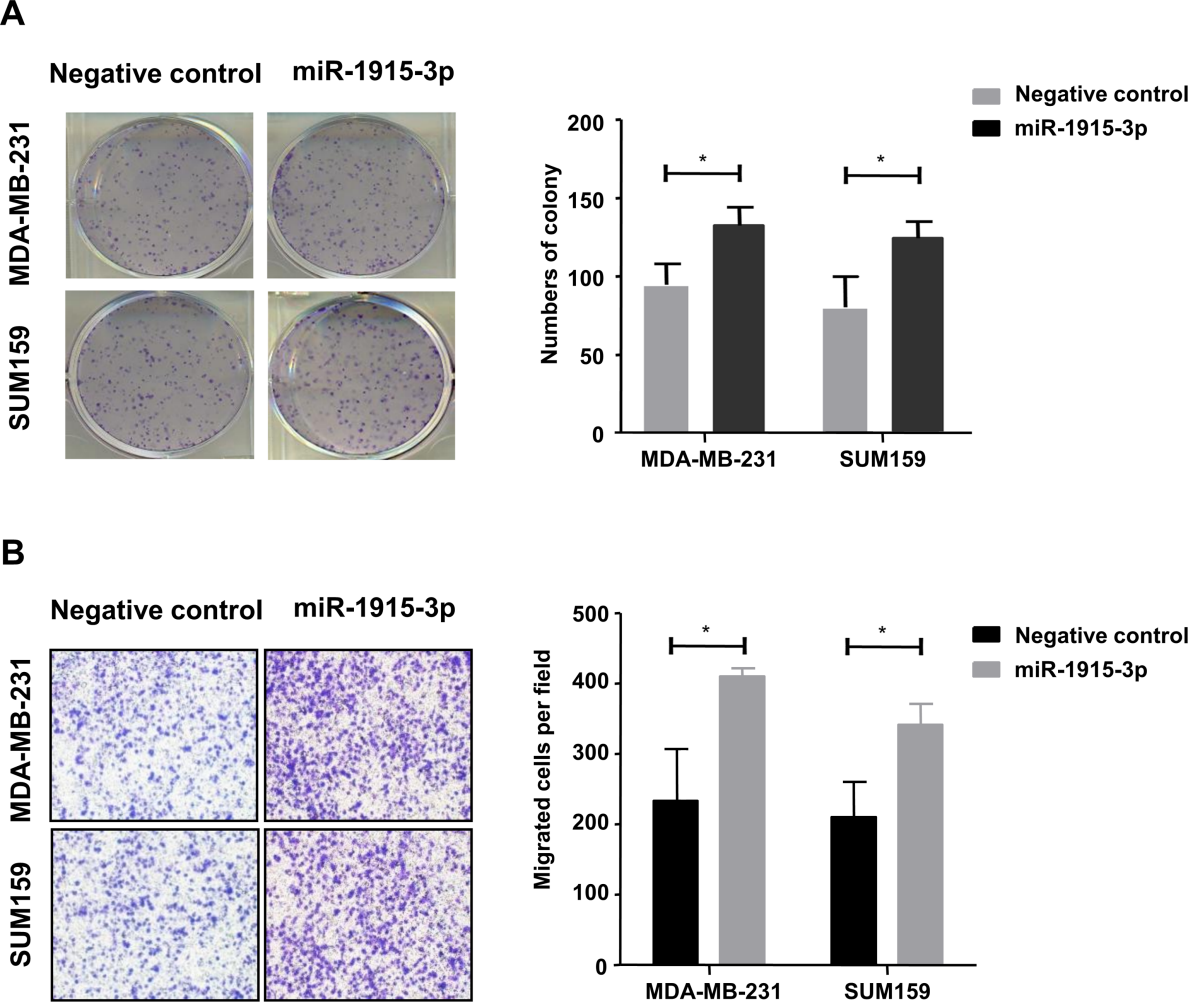
miR-1915-3p promoted breast cancer cell clone formation and migration in vitro. (A) Clone formation detection was performed on both MDA-MB-231 and SUM-159 cells which were both transfected with miR-1915-3p, statistic results were shown on the right. (B) Migration capacity of MDA-MB-231 and SUM-159 tumor cells transfected with miR-455-3p mimic or negative control was detected by using the Transwell assay. Data was shown as mean±s.e.m. from three independent experiments (*P<0.05).

To further confirm the above data, similar results were proved by colony formation assay on both cell lines (Fig 5A). The over-expressed miR-1915-3p group formed more clones than the control group, and this result reached statistically difference.

### miR-1915-3p promotes the migration of MDA-MB-231 and SUM-159 cells

MDA-MB-231 and SUM-159 cells over-expressing miR-1915-3p were used for cell migration assay. As shown in Fig 5B, transwell assay demonstrated the number of miR-1915-3p overexpressed group cells passing through the inserts was significantly more than the negative control group, which indicated the migration ability of breast cancer cells were promoted by miR-1915-3p.

### miR-1915-3p activated ERK1/2 through repression of DUSP3 expression

**Fig 6:**
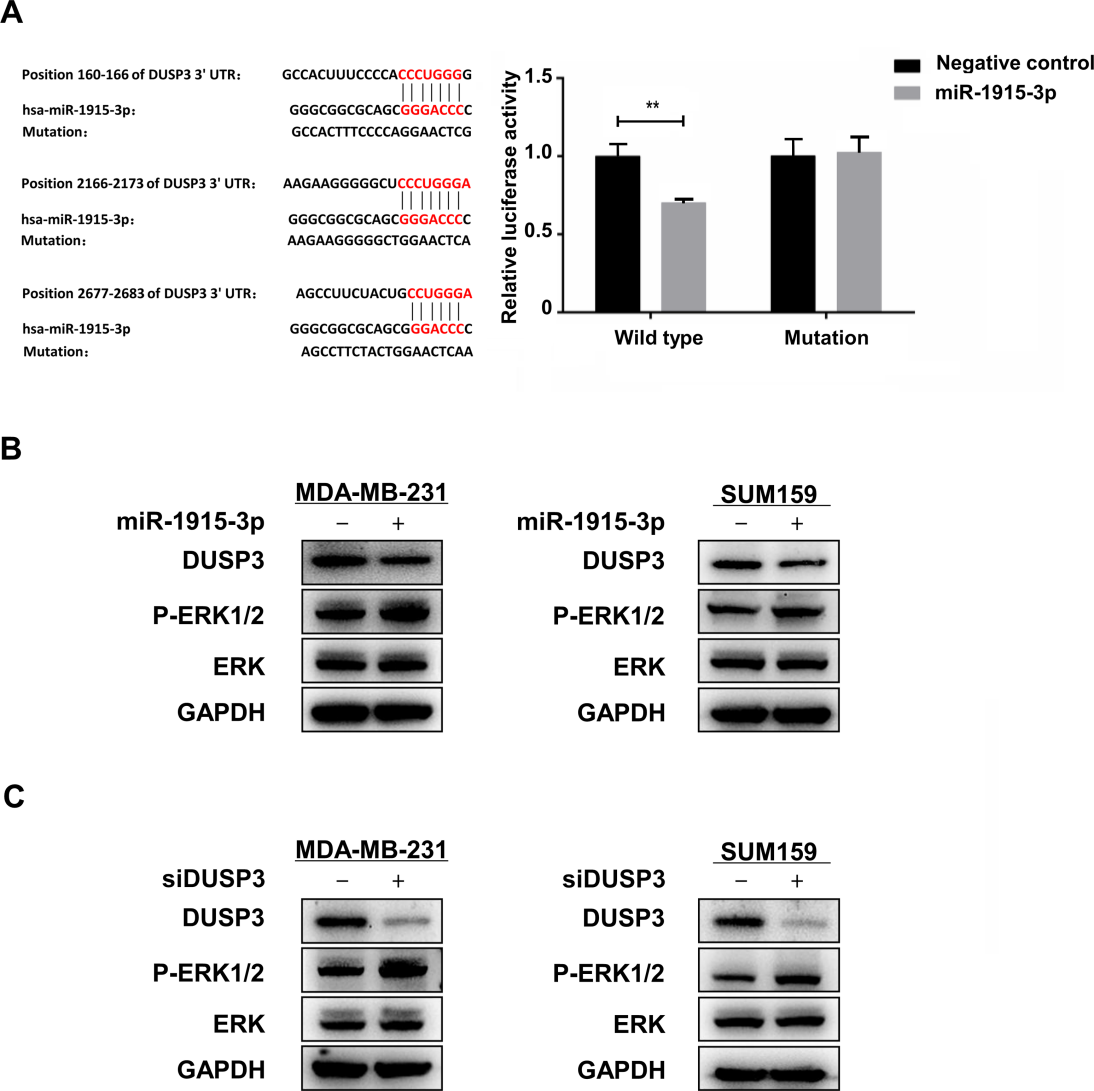
miR-1915-3p activated ERK1/2 through repression of DUSP3 expression. The wild type or mutant psiCHECK2-DUSP-3’UTR plasmid and miR-1915-3p or miR-NC were co-transfected into 293FT cells respectively, and luciferase activity was analyzed in (A). (B) Expression of DUPS3 and phospho-ERK protein was detected in MDA-MB-231 and SUM-159 cells transfected with miR-455-3p mimics or negative control using Western blot. (C) Expression of DUPS3 and phospho-ERK protein was detected in MDA-MB-231 and SUM-159 cells transfected with DUSP3 siRNA or negative control using Western blot.

To explore the mechanism of miR-1915-3p regulating the tumor growth and metastasis, we searched cancer-related targets of miR-1915-3p on bioinformatics predicting software Targetscan. We found that there were three potential binding sits on the 3’-untranslated region (3’UTR) of dual-specificity phosphatase 3 (DUSP3). We performed the dual luciferase reporter assay to explore whether DUSP3 was regulated by miR-1915-3p directly. 3’UTR of DUSP3 containing wild type or mutant miR-1915-3p target sequences was cloned into the psiCHECK2 plasmids. The plasmids and miR-1915-3p or miR-NC were co-transfected into 293FT cells respectively, and luciferase activity was analyzed. The results showed that miR-1915-3p decreased the luciferase activity of the wild type psiCHECK2-DUSP3-3’UTR plasmid, but had no effect on the mutant plasmid (Fig 6A). Western Blot results also showed that DUSP3 protein expression was downregulated in miR-1915-3p mimic groups of MDA-MB-231 and SUM-159 (Fig 6B). DUSP3 is a phosphatase which has been reported to dephosphorylates ERK1/2 in various cells. RNAi knockdown experiments indicated that repression of DUSP3 expression could increase the level of ERK1/2 phosphorylation in MDA-MB-231 and SUM-159 cells (Fig 6C). Overexpression of miR-1915-3p in MDA-MB-231 and SUM-159 decreased the expression of DUSP3 and activated ERK1/2 (Fig 6B). These results suggested that DUSP3 was a target of miR-1915-3p and miR-1915-3p might activate ERK1/2 through repression of DUSP3 expression.

## Discussion

miRNAs are closely associated with the carcinogenesis of diverse tumor types which are viewed as oncogenes or tumor repressor genes[13–16]. With the analysis of miRNAs in tumor expression profiles, extensive researches on malignant tumors have shown that miRNAs, both in solid tumors and in blood tumors, were quite different from those in normal tissues from where they originated[17, 18]. In addition, the expression profiles of miRNAs in different tumor types are also quite different[19]. Dysregulated expression of miRNAs in tumors secreting into blood actively or passively, might change the serum level of miRNAs[12], which provides an opportunity to diagnose tumors and predict progress.

The circulating miRNAs have already been verified and applied as biomarkers for diagnosis and prognosis in various cancers[20–23]. In our study, serum groups were mixed to perform Affymetrix miRNA profiling array so that the intra-group error could be minimized. Then, to reduce inter-group error, a Taqman probe assay was used for quantitative analysis on single serum miRNA of patients, and different grouping and merging methods were taken so that the selected miRNAs could have increased probability of inter-group differences. However, the results of validation did not coincide with the miRNA array which meant the mixed serum might not reflect the situation of each patient accurately, at least in our case. But still, miR-455-3p and miR-1915-3p showed value to serve as a diagnostic biomarkers of breast cancer. We found that the serum level of miR-1915-3p was upregulated and that miR-455-3p was downregulated in breast cancer patients compared with healthy volunteers which showed that the miR-1915-3p and miR-455-3p might serve as diagnostic markers of breast cancer. Furthermore, the patients with lymph node metastasis had a higher level of serum miR-1915-3p and lower level of serum miR-455-3p than those patients without lymph node metastasis. Patients with infiltrating carcinoma showed the same trends than those with carcinoma in situ. ROC curve is a well recognized statistical method and is widely used for identifying diseases predictive accuracy. In this study, both of the two miRNAs exhibited great reliability in predicting lymph node metastasis and infiltrating status of breast cancer in ROC curve analysis. These results showed that miR-1915-3p and miR-455-3p might have the potential to predict breast cancer progress. Considering the number of serum samples in each group, it was still required to carry out further experiments with enlarged sample size to verify these results.

miR-1915-3p is over-expressed in certain human breast cancer cells. We verified that miR-1915-3p could promote the migration and proliferation of breast cancer cells, which showed their association with disease progress. DUSP3 was further proved to be a direct target gene of miR-1915-3p by Dual-luciferase report system assays and Western blot. DUSP3, a member of the DUSP family of phosphatases, could directly dephosphorylate ERK1/2 in various cells. Repression of DUSP3 expression increased the phospho-ERK1/2 levels in HeLa cells[24], non–small cell lung cancer cells(H1792 and H460)[25] and breast cancer cells(MCF-7 and SKBR3)[26]. We demonstrated that downregulation of DUSP3 in MDA-MB-231 or SUM159 could also enhance the phosphorylation of ERK which might be one of the mechanism that miR-1915-3p regulates the tumor cells. It has been widely recognized that ERK signaling is participated in the progression and metastasis of various tumors[27–29]. Activated ERK phosphorylates a number of substrates, such as other kinases and transcription factors, which execute pro-tumor programs related to cell cycle progression, metastasis, and evasion from cell death[30].

In summary, our study demonstrated that miR-1915-3p might promote the proliferation and metastasis of breast cancer by repression of DUSP3 and serum miR-1915-3p and miR-455-3p could serve as diagnostic and predictive biomarkers for breast cancer, which might be adopted for the large scale clinical diagnosis in the future.

## Supporting information

S1 Fig Excluded seven genes expression level of individual serum sample in diagnosis set. (TIF)

S1 Table Demographic and clinical characteristics of the patients and control individuals in this study. (DOCX)

S1 File Statistical information. (XLSX)
