# Supplementary figures and images for: Identification of serum miR-1915-3p and miR-455-3p as biomarkers for breast cancer
Source: PLoS One. 2018 Jul 26;13(7):e0200716. doi: 10.1371/journal.pone.0200716 (PMC6062026; doi:10.1371/journal.pone.0200716)

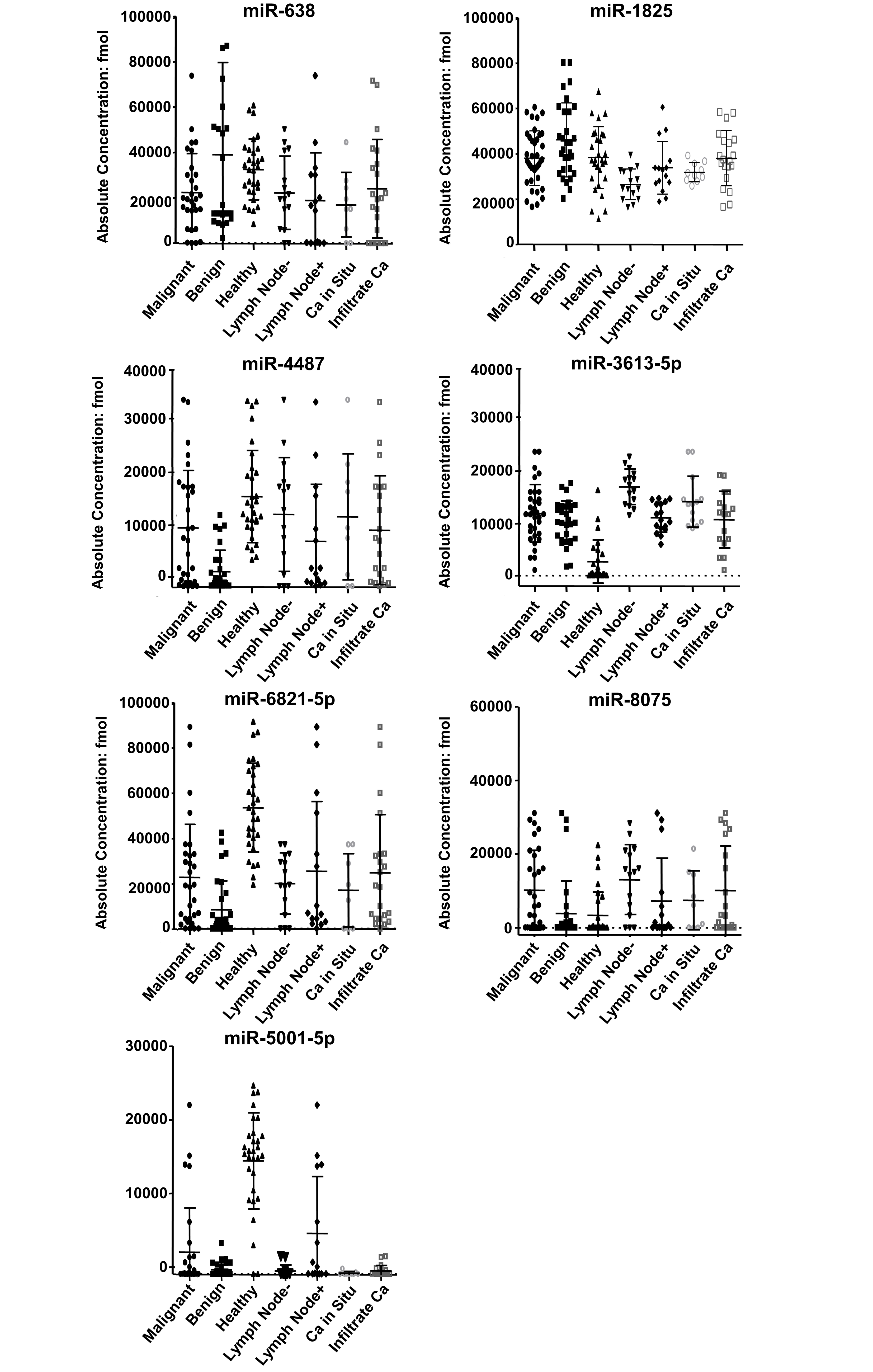

Supplement: S1 Fig — (TIF) [file pone.0200716.s001.tif]
