# Supplementary material for: Identification of serum miR-1915-3p and miR-455-3p as biomarkers for breast cancer
Source: PLoS One. 2018 Jul 26;13(7):e0200716. doi: 10.1371/journal.pone.0200716 (PMC6062026; doi:10.1371/journal.pone.0200716)
Supplement: S1 Table — (DOCX) [file pone.0200716.s002.docx]

|  | **tumor** | **benign** | **healthy** |
| --- | --- | --- | --- |
| **n** | 94 | 100 | 100 |
| **age** |  |  |  |
| **age group** |  |  |  |
| **≤40** | 8(8.4%) | 59(59%) | 38(38%) |
| **>40** | 86(91.5%) | 41(41%) | 62(62%) |
| **Histological subtype** |  |  |  |
| **Invasive lobular carcinoma** | 17(18.1%) |  |  |
| **Invasive ductal carcinoma** | 73(77.7%) |  |  |
| **Others** | 4(4.2%) |  |  |
| **TNM stage** |  |  |  |
| **0** | 15(16.0%) |  |  |
| **I** | 30(31.9%) |  |  |
| **II** | 39(41.5%) |  |  |
| **III** | 7(7.4%) |  |  |
| **IV** | 3(3.2%) |  |  |
| **molecular subtype** |  |  |  |
| **Luminal A** | 33(35.1%) |  |  |
| **Luminal B** | 39(41.5%) |  |  |
| **Basal like** | 15(16.0%) |  |  |
| **Her2 overexpressed** | 7(7.4%) |  |  |
| **Family history of BrCa** |  |  |  |
| **Yes** | 4(4.3%) |  |  |
| **No** | 90(95.7%) |  |  |
| **Pre-menopausal** | 52(55.3%) | 89(89%) | 54(54%) |
| **Post-menopausal** | 42(44.7%) | 11(11%) | 46(46%) |
| **Lymph nodes** |  |  |  |
| **positive** | 36(38.3%) |  |  |
| **(1-4)** | 11(11.7%) |  |  |
| **(5-above)** | 25(26.6%) |  |  |
| **negative** | 58(61.7%) |  |  |
